# Exploring the effect of network topology, mRNA and protein dynamics on gene regulatory network stability

**DOI:** 10.1038/s41467-020-20472-x

**Published:** 2021-01-08

**Authors:** Yipei Guo, Ariel Amir

**Affiliations:** 1 John A. Paulson School of Engineering and Applied Sciences, Harvard University, Cambridge, MA USA; 2 Program in Biophysics, Harvard University, Boston, MA 02115 USA

**Keywords:** Dynamical systems, Regulatory networks, Complex networks

## Abstract

Homeostasis of protein concentrations in cells is crucial for their proper functioning, requiring steady-state concentrations to be stable to fluctuations. Since gene expression is regulated by proteins such as transcription factors (TFs), the full set of proteins within the cell constitutes a large system of interacting components, which can become unstable. We explore factors affecting stability by coupling the dynamics of mRNAs and proteins in a growing cell. We find that mRNA degradation rate does not affect stability, contrary to previous claims. However, global structural features of the network can dramatically enhance stability. Importantly, a network resembling a bipartite graph with a lower fraction of interactions that target TFs has a higher chance of being stable. Scrambling the *E. coli* transcription network, we find that the biological network is significantly more stable than its randomized counterpart, suggesting that stability constraints may have shaped network structure during the course of evolution.

## Abstract (web summary)

Maintaining protein expression levels is essential to cellular homeostasis. Here, the authors investigate how transcription factors affect the stability of protein expression in a gene regulatory network, and highlight the importance of network topology.

## Introduction

Cells require different protein levels to survive in different external environments. The expression of these proteins within the cell are therefore highly regulated. An important regulatory mechanism involves transcription factors (TFs), which are themselves proteins that can either up or downregulate the transcription of mRNAs coding for other proteins by binding to enhancer or promoter regions of the regulated gene^1^. Despite the importance of maintaining desired protein concentrations within cells, factors affecting the stability of these concentrations to perturbations have received little attention.

One approach of studying the stability of such systems with a large number of interacting components was introduced by May in the 1970s in the context of complex ecological communities^2^. The idea is that in a *n*-species community, the dynamics of the abundances *N*_*i*_ of each species may in general be described by a set of ordinary differential equations:

1 $$\frac{\mathrm{d}{N}_{i}}{\mathrm{d}t}={f}_{i}({N}_{1},{N}_{2},...{N}_{n})$$

for *i* = 1, 2, ..., *n*, with corresponding steady-state solution ${N}_{i}^{ss}$ such that ${f}_{i}({\overrightarrow{{\bf{N}}}}^{ss})=0$ ∀ *i*. The dynamics of small perturbations about this steady-state ${x}_{i}(t)={N}_{i}(t)-{N}_{i}^{ss}$, when linearized about ${N}_{i}^{ss}$, has the form:

2 $$\frac{\mathrm{d}\overrightarrow{{\bf{x}}}}{\mathrm{d}t}={\bf{A}}\overrightarrow{{\bf{x}}},$$

where **A** is the Jacobian matrix with elements ${A}_{ij}={\left(\frac{\partial {f}_{i}}{\partial {N}_{j}}\right)}^{ss}$. If all the eigenvalues of **A** have a negative real part, the system relaxes back to the steady-state upon perturbations and the steady-state is said to be stable; if any of the eigenvalues have a positive real part, the steady-state is unstable as the system will move away from it (exponentially fast) when infinitesimally perturbed. To construct **A**, one would need to precisely know the functions *f*_*i*_, which is often hard to obtain. May’s approach was to model **A** as a random matrix with independent, identically distributed off-diagonal elements (with mean 0, standard deviation *σ*, and fraction of non-zero elements *C*) and constant diagonal elements—*a*. In the context of ecology, *σ* reflects the average interaction strength between species, *C* is the density of interactions or the probability that any two species interact, while *a* is the self-regulation term which sets the relaxation time-scale of the system if there were no other pairwise interactions. From random matrix theory (RMT) and in particular the circular law for matrix eigenvalue distributions^3,4^, this system is stable if and only if $a \, > \, \sigma \sqrt{nC}$. This implies that the system becomes unstable above some critical size, and that increasing *a* stabilizes the system and allows for stronger interactions between species.

This approach has also been used to analyze other large interacting systems. In particular, it has been used to argue why weak repressions by microRNAs, thought of as effectively increasing the degradation rate of mRNAs, confer stability to gene regulatory networks^5,6^. However, such a framework does not take into account the functional form of *f*_*i*_ and in particular that the matrix elements often depend on the steady-state solutions themselves. These details of the model can be important—for example, when competition for resources between ecological species are explicitly modeled (using MacArthur’s consumer-resource model), even when the interactions (i.e., preferences of each species for the different resources) are completely random, the spectrum of the Jacobian matrix that represents effective pairwise interaction between species is no longer circular (but rather, follows the Marchenko-Pastur distribution)^7^. Furthermore, transcriptional regulatory networks are not random but instead have distinct structural features. The structure of interaction networks has been known to affect stability in other models^7–10^. However, how these features affect the stability of gene regulatory networks has not been explored.

Here, by analyzing a model that takes into account the transcription of mRNAs from genes, translation of mRNAs into proteins, and transcriptional regulation by proteins, we investigate the stability of this large system of coupled mRNAs and proteins in growing cells and find that while the mRNA degradation rate can affect relaxation rate back to steady-state levels, it does not affect whether the system is stable. Instead, stability can depend strongly on the global structural features of the interaction network. In particular, given the same number of proteins, TFs, number of interactions, and regulation strengths, a network with a lower fraction of interactions that target TFs has a higher chance of being stable. In the limit where there are no TF–TF interactions i.e. all TFs regulate proteins that are not TFs, it is possible for the system to remain stable for arbitrarily large system sizes, unlike random networks which become unstable when system size becomes too large. By scrambling the *E. coli*. transcription network, we find that the topology of real networks can stabilize the system since the randomized network with the same number of regulatory interactions is often unstable. These findings suggest that constraints imposed by system stability may have played a significant role in shaping the existing regulatory network during the evolutionary process. By carrying out the analysis for different physiological states the cell can be in (corresponding to different sets of dynamical equations) and with different choices of parameter distributions, we also show that our main results and conclusions are robust to the details of the model.

## Results

### The model

**Fig. 1:**
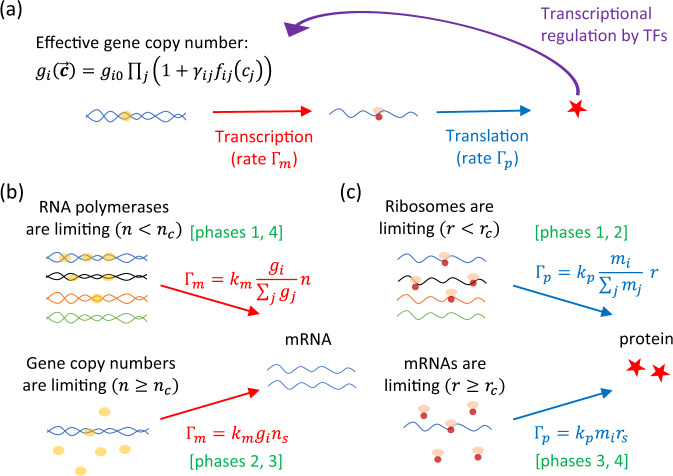
Schematic illustration of the gene expression model. **a** The dynamics of protein and mRNA concentrations are coupled through transcriptional regulation, where some of the proteins (e.g., transcription factors) modulate the effective gene copy numbers *g*_*i*_ and hence the transcription rate of other genes. **b** If RNA polymerase is in excess, transcription rate Γ_*m*_ of gene *i* is proportional to its effective gene copy number *g*_*i*_. If instead RNA polymerase is limiting, Γ_*m*_ is proportional to the gene allocation fraction *ϕ*_*i*_ = *g*_*i*_/∑_*j*_*g*_*j*_. **c** Translation rate Γ_*p*_ is proportional to mRNA number *m*_*i*_ if mRNAs are limiting, and proportional to the mRNA fraction *m*_*i*_/∑_*j*_*m*_*j*_ if ribosomes are limiting. There are four different phases of the model depending on whether RNA polymerases and ribosomes are limiting.

Gene expression involves two major steps: transcription and translation (Fig. 1a). Transcription is the process in which mRNA is synthesized by RNA polymerase using DNA as a template. The transcription rate of a gene *i* therefore depends on the number of RNA polymerases *n* and its effective gene copy number *g*_*i*_ which takes into account both its copy number and how strongly RNA polymerase can bind to the promoter of that gene^11^. Due to the presence of TFs, ${g}_{i}(\overrightarrow{{\bf{c}}})$ can in general depend on the set of protein concentrations $\overrightarrow{{\bf{c}}}$ (Fig. 1a). We assume that multiple TFs acting on the same gene act independently, with their effects stacking multiplicatively. This allows for both OR- and AND-gate-like combinatorial effects^12^, and can emerge from a thermodynamic model of TF binding (Supplementary Note 1). Therefore, we adopt the following form for transcriptional regulation throughout the paper:

3 $${g}_{i}(\overrightarrow{{\bf{c}}})={g}_{i0}\prod_{j}(1+{\gamma }_{ij}{f}_{ij}({c}_{j})),$$

where *g*_*i*0_ is the effective gene copy number of *i* if it were unregulated (randomly drawn from a uniform distribution), and *γ*_*ij*_ controls the type and strength of regulation, i.e., how much gene expression of *i* changes in the presence of the TF *j*. In particular, *γ*_*ij*_ > 0 if *j* upregulates *i* and −1 ≤ *γ*_*ij*_ < 0 if *j* downregulates *i*. For each regulatory interaction, we assume that the fold-change Ω_*ij*_ is drawn from a uniform distribution between 1 and ${{{\Omega }}}_{\max }$, such that

4 $${\gamma }_{ij}=\left\{\begin{array}{ll}{\Omega }_{ij}-1&{\rm{if}}\,{\gamma }_{ij} \, > \, 0\,({\rm{upregulating}})\\ \frac{1}{{\Omega }_{ij}}-1&{\rm{if}}\,{\gamma }_{ij} \, < \, 0\,({\rm{downregulating}})\end{array}\right.$$

since this would allow *g*_*i*_(*c*_*j*_) to increase (if *j* upregulates *i*) or decrease (if *j* downregulates *i*) by a factor of Ω_*ij*_ in the limit of high *c*_*j*_. In Supplementary Note 5, we show that the main results do not depend on the particular distribution *P*(Ω) used.

Motivated by experimental measurements of the relationship between TF input and gene expression output showing a sigmoidal functional form of *f*_*ij*_(*c*_*j*_)^13,14^, we take it to be a Hill function

5 $${f}_{ij}({c}_{j})=\frac{{c}_{j}^{h}}{{K}_{ij}^{h}+{c}_{j}^{h}},$$

with the Hill coefficient *h* > 0.

Following ref. ^11^, we assume a threshold number *n*_*c*_ of RNA polymerases above which the gene copy number is limiting the transcription rate (Fig. 1b). When this is the case, the transcription rate is proportional to *g*_*i*_ and is independent of *n*. If instead *n* < *n*_*c*_, it is the RNA polymerases that are limiting, in which case the different genes have to compete for the limited pool of RNA polymerases. The transcription rate of a gene *i* is then proportional to both *n* and the fraction of RNA polymerases working on that gene, the gene allocation fraction:

6 $${\phi }_{i}(\overrightarrow{{\bf{c}}})=\frac{{g}_{i}(\overrightarrow{{\bf{c}}})}{{\sum }_{j}{g}_{j}(\overrightarrow{{\bf{c}}})}.$$

Denoting the number of different genes by *N*, the dynamics of mRNA *m*_*i*_ for *i* = 1, ...*N* can therefore be described by the following equation:

7 $$\frac{\mathrm{d}{m}_{i}}{\mathrm{d}t}=\left\{\begin{array}{ll}{k}_{m}{\phi }_{i}(\overrightarrow{{\bf{c}}})n-\frac{{m}_{i}}{{\tau }_{m}}&{\rm{if}}\,n \, < \, {n}_{c}\\ {k}_{m}{g}_{i}(\overrightarrow{{\bf{c}}}){n}_{s}-\frac{{m}_{i}}{{\tau }_{m}}&{\rm{if}}\,n\ge {n}_{c}\end{array}\right.$$

where *k*_*m*_ characterizes the transcription rate of a single RNA polymerase, *τ*_*m*_ is the mRNA lifetime, and *n*_*s*_ is the maximum number of RNA polymerases per gene.

Similarly for the process of translation where ribosomes make proteins using mRNA as a template, the translation rate depends on the number of ribosomes *r* and the mRNA copy number *m*_*i*_. As for RNA polymerases, there is also a threshold number of ribosomes *r*_*c*_ above which mRNA number is limiting and below which ribosomes are limiting (Fig. 1c). The dynamics of protein numbers _*pi*_ for *i* = 1, ..., *N*, with *p*_*N*−1_ = *n* corresponding to RNA polymerases and *p*_*N*_ = *r* corresponding to ribosomes, are therefore given by:

8 $$\frac{\mathrm{d}{p}_{i}}{\mathrm{d}t}=\left\{\begin{array}{ll}{k}_{p}\frac{{m}_{i}}{{\sum }_{j}{m}_{j}}r-\frac{{p}_{i}}{{\tau }_{p}}&{\rm{if}}\,r \, < \, {r}_{c}\\ {k}_{p}{m}_{i}{r}_{s}-\frac{{p}_{i}}{{\tau }_{p}}&{\rm{if}}\,r\ge {r}_{c}\end{array}\right.,$$

where *k*_*p*_ characterizes the translation rate of a single ribosome, *τ*_*p*_ is the protein lifetime, and *r*_*s*_ is the number of ribosomes per mRNA when ribosomes are in excess.

Depending on whether the RNA polymerases and ribosomes are limiting, there are four different cellular phases (Fig. 1b, c). The regime where *n* ≥ *n*_*c*_ and *r* ≥ *r*_*c*_ (phase 3 of the model, where the production rate of mRNAs and proteins are proportional to gene and mRNA copy numbers respectively) has been widely studied^15–17^, but has been shown to be inconsistent with experimental observations in wild-type cells showing the exponential growth of protein levels^18,19^. Instead, the regime where *n* < *n*_*c*_ and *r* < *r*_*c*_ (phase 1 of the model) is the one where wild-type fission yeast^18^ and mammalian cells appear to be in^19^. We therefore focus on this phase for the rest of the paper. Note, however, that the phase 3 regime has been experimentally observed in defective budding yeast and mammalian cells that are excessively large^20^, whereas the regime where RNA polymerases are in excess (*n* ≥ *n*_*c*_) while ribosomes are limiting (*r* < *r*_*c*_) (phase 2 of the model) has been observed in mutant fission yeast^18^. We will address these two phases in the SI. The regime where *n* < *n*_*c*_ and *r* ≥ *r*_*c*_ (phase 4 of the model) is biologically unrealistic as ribosomes are typically more expensive to make compared to other proteins and hence having excess ribosomes while RNA polymerases are limited would be inefficient^21,22^. This regime is therefore not considered.

It will be convenient to consider the dynamics of the concentrations of mRNAs ${c}_{mi}=\frac{{m}_{i}}{V}$ and proteins ${c}_{i}=\frac{{p}_{i}}{V}$. In bacteria^23,24^ and mammalian cells^25^, the volume of the cell *V* is approximately proportional to the total protein mass. Hence, we assume for simplicity that each protein has the same mass and set the cell density to be 1, such that *V* = ∑_*i*__*pi*_. The dynamics for concentrations in phase 1 are then given by:

9 $$\frac{\mathrm{d}{c}_{mi}}{\mathrm{d}t}={k}_{m}{\phi }_{i}(\overrightarrow{{\bf{c}}}){c}_{n}-{c}_{mi}\left({k}_{p}{c}_{r}+\frac{1}{\tau }\right),$$

10 $$\frac{\mathrm{d}{c}_{i}}{\mathrm{d}t}={k}_{p}{c}_{r}\left(\frac{{c}_{mi}}{{c}_{mT}}-{c}_{i}\right),$$

where *c*_*mT*_ = ∑_*i*_*c*_*mi*_ is the total concentration of all mRNAs and $\frac{1}{\tau }=\frac{1}{{\tau }_{m}}-\frac{1}{{\tau }_{p}}$ is the difference between mRNA and protein degradation rates (which can be positive or negative). A summary of the list of model parameters can be found in Supplementary Table 1.

While these equations govern the dynamics of average concentrations and hence do not capture stochastic effects inherent in gene expression and in the binomial sampling of molecules during cell division, these fluctuations do not affect the average steady-state concentrations if the number of molecules is large (see Supplementary Note 2, Supplementary Fig. 1). In fact, these fluctuations can be considered as perturbations about steady-state values, and we investigate the stability of the system to such perturbations in the rest of the paper.

### Effects of network features and topology on stability of the system

To study how properties of the transcriptional regulatory network affect the stability of the system, we first consider the regime where the lifetime of mRNAs is much shorter than that of proteins, which is typically true for wild-type cells^26^. In this limit of fast mRNA degradation, the relaxation dynamics of mRNA is much faster than that of proteins such that $\frac{\mathrm{d}{c}_{mi}}{\mathrm{d}t}\approx 0$ at all times. Eliminating the fast process (by substituting the steady-state mRNA concentrations ${c}_{mi}=\frac{{k}_{m}{c}_{n}}{{k}_{p}{c}_{r}+\frac{1}{\tau }}{\phi }_{i}(\overrightarrow{{\bf{c}}})$ obtained from Eq. (9) into Eq. (10)), the dynamics of protein concentrations can be written as a set of *N* ODEs:

11 $$\frac{\mathrm{d}{c}_{i}}{\mathrm{d}t}\approx {k}_{p}{c}_{r}\left({\phi }_{i}(\overrightarrow{{\bf{c}}})-{c}_{i}\right).$$

The stability of the system therefore depends only on the eigenvalues of the *N* × *N* Jacobian matrix ${\bf{A}}={k}_{p}{c}_{r}^{ss}({\bf{M}}-{\bf{I}})$, where we define the interaction matrix

12 $${M}_{ij}=\frac{\partial {\phi }_{i}}{\partial {c}_{j}}{| }_{\overrightarrow{{\bf{c}}} = {\overrightarrow{{\bf{c}}}}^{ss}},$$

with the steady-state protein concentrations given by ${c}_{i}^{ss}={\phi }_{i}({\overrightarrow{{\bf{c}}}}^{ss})$ (from Eq. (11)).

Denoting *λ*_*M*_ as the eigenvalues of **M**, the system is stable as long as the maximal real part of these eigenvalues ${\lambda }_{M,{r}_{\max }}$ is smaller than 1 (such that all eigenvalues of **A** have a negative real part). It is therefore useful to understand the structure of **M** by breaking it into two parts using Eq. (6):

13 $${M}_{ij}={c}_{i}^{ss}({M}_{1,ij}-{M}_{2,ij}),$$

where

14 $${M}_{1,ij}=\frac{\partial \mathrm{log}\,{g}_{i}}{\partial {c}_{j}}$$

captures the direct interactions between proteins, while

15 $${M}_{2,ij}=\frac{\partial \mathrm{log}\,{g}_{T}}{\partial {c}_{j}}=\sum_{k}{c}_{k}^{ss}\frac{\partial \mathrm{log}\,{g}_{k}}{\partial {c}_{j}}$$

is a rank-1 matrix that captures the indirect interactions arising from competition for ribosomes.

It can be shown that both the structure of **M** (Eq. (13)) and the fact that stability only depends on **M** still hold in the other phases, despite the exact equations for protein dynamics being different (see Supplementary Note 3). Therefore, even though the simulations in the rest of this section are carried out in phase 1, our findings and conclusions also apply to the other phases.

#### Stability of the system scales with $\sqrt{N}$ for random regulatory networks

We start by exploring the stability of fully random regulatory networks, which we take to be our null model.

Since the maximum eigenvalue of a random matrix depends on the standard deviation of its elements, we first carry out a naive estimate of how the elements of **M** scale with *N*. With *g*_*i*_(*c*) given by Eq. (3),

16 $$\frac{\partial \mathrm{log}\,{g}_{i}}{\partial {c}_{j}}=\frac{{\gamma }_{ij}}{1+{\gamma }_{ij}{f}_{ij}({c}_{j})}\frac{\partial {f}_{ij}}{\partial {c}_{j}}.$$

Biologically, TF concentrations are often comparable to the values of dissociation constants *K*_*d*_ for DNA binding^26^. Therefore, since *c*_*j*_ ~ 1/*N*, we also choose *K*_*ij*_ ~ 1/*N* (Eq. (5)), which would allow cells to maintain the full range of gene expression response. From Eq. (5), this implies that *f*_*ij*_ ~ *O*(1) and $\frac{\partial {f}_{ij}}{\partial {c}_{j}} \sim N$, and hence *M*_1_ and *M*_2_ also scale with *N* (Eqs. (14), (15)). We therefore expect *M*_*ij*_ ~ *O*(1) (Eq. (13)), and hence (from RMT), for ${\lambda }_{M,{r}_{\max }}$ to scale approximately as $\sqrt{N}$ for random interaction networks. ${\lambda }_{M,{r}_{\max }}$ also increases with the strength of the interactions *γ*, implying that the system will become unstable either when *N* exceeds a critical number or the regulation strength becomes too high. However, this argument neglects correlations between the elements of **M**, which could potentially be relevant. In fact, we will see in the later sections that the structure of **M** (Eq. (13)) plays an important role in influencing the stability of the system.

Therefore, to test if this scaling relation holds, we constructed networks of a specified interaction density *ρ* by randomly selecting *ρN*^2^ interactions from the *N*(*N* − 1) possibilities (where we have assumed that ribosomes cannot act as TFs), and choose half of the interactions to be upregulating with the remaining half being downregulating.

**Fig. 2:**
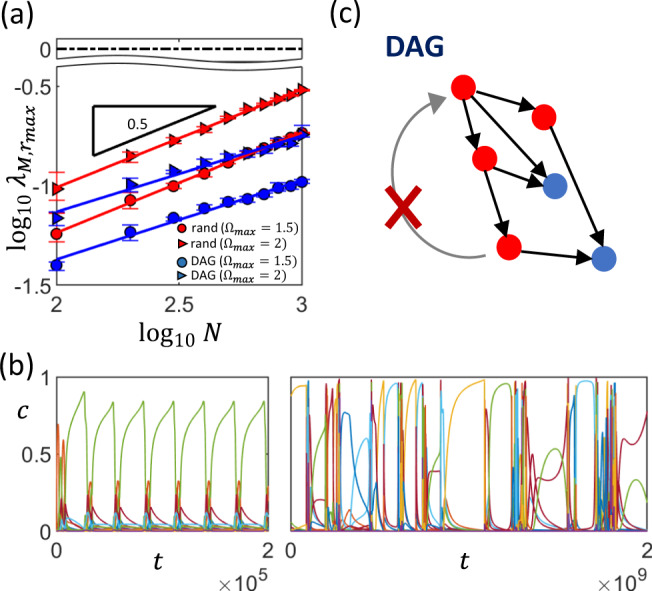
Stability of random interaction networks. **a** For random interaction networks (red markers,`rand'), the maximal real part of the eigenvalues of the interaction matrix ${\lambda }_{M,{r}_{\max }}$ scales with $\sqrt{N}$. Surprisingly, for random directed acyclic networks (blue markers,`DAG'), ${\lambda }_{M,{r}_{\max }}$ also scales approximately with $\sqrt{N}$. In both of these cases, increasing the interaction strength from ${{{\Omega }}}_{\max }=1.5$ (circles) to ${{{\Omega }}}_{\max }=2$ (triangles) increases ${\lambda }_{M,{r}_{\max }}$. These results suggest that the system will become unstable (i.e., ${\mathrm{log}\,}_{10}({\lambda }_{M,{r}_{\max }})$ exceeds 0, indicated by the black dashed line) when *N* or ${{{\Omega }}}_{\max }$ becomes too large. Each data point is obtained from an average of 10 randomly drawn networks, with error bars indicating the interquartile range. Each random network is constructed by randomly selecting *ρN*^2^ interactions from *N*(*N* − 1) possibilities, with half of the interactions chosen to be upregulating and the remaining half to be downregulating. The construction of DAGs is described in (**c**). For each regulatory interaction, fold change is chosen uniformly between 1 and ${{{\Omega }}}_{\max }$. [Other parameters: *ρ* = 0.01, *h* = 1]. **b** When systems go out of stability, dynamics of protein concentrations *c* exhibit oscillatory (left, ${{{\Omega }}}_{\max }=20$) followed by chaotic behavior (right, ${{{\Omega }}}_{\max }=200$) as interaction strengths are increased. [Other parameters: *N* = 200, *ρ* = 0.2, *h* = 1, fully random network, time *t* is in units of 1/*k*_*p*_.] **c** Random directed acyclic networks are constructed by randomly drawing connections between proteins (red circles represent TFs, blue circles represent non-TFs). If a drawn connection creates a loop (e.g., the gray arrow with a cross on it), it is rejected.

By taking the ensemble average over the randomly drawn networks, we indeed recover the $\sqrt{N}$ scaling (Fig. 2a), which is also robust to the fraction of up- and downregulatory interactions (see Supplementary Note 4, Supplementary Fig. 2a) and the distribution of fold-changes *P*(Ω) (see Supplementary Note 5, Supplementary Fig. 3). For sufficiently large *N* or ${{{\Omega }}}_{\max }$, we can no longer find the fixed point of the system. Nevertheless, by simulating the dynamics, we find that for interaction networks of a given *N* and *ρ*, we get oscillatory, followed by chaotic behavior as ${{{\Omega }}}_{\max }$ is increased (Fig. 2b). Similar phenomena have also been described and analyzed in models of neural networks^27^ and ecological systems^28^. While certain biochemical circuits have been known to generate oscillations such as in the cell cycle and the circadian clock, the oscillatory dynamics observed here is of a different nature—it does not come about from any specific fine-tuning of the network but, rather, emerges from having a large number of randomly and strongly interacting genes.

However, transcriptional regulatory networks are typically not random. Instead, they are enriched for distinct structural features such as the following motifs: feedforward loops (FFL), single-input module (SIM), and dense overlapping regulons (DOR) which do not contain any loops besides autoregulatory ones^1,29^. In the next few subsections, we therefore explore the effects of network topology on system stability.

#### Random directed acyclic networks can also be unstable

Since transcription networks as a whole resemble directed acyclic graphs (DAGs)^1,29^, we explore the stability of such networks.

In systems where the Jacobian matrix reflects the presence of direct interactions between components, the elements of the Jacobian matrix *A*_*ij*_ is 0 if *j* does not influence or regulate *i*. In such cases, if there are no interaction loops involving 2 or more components (e.g., E regulates F which also regulates E), **A** can be written as a triangular matrix for such a DAG and the eigenvalues are the diagonal elements of the matrix, i.e., the self-regulation loops. The system is therefore stable if there are no auto-activation among the components, i.e., there are no positive elements along the diagonal of **A**.

In our case, the presence of indirect interactions captured by the additional **M**_**2**_ matrix (Eq. (13)) implies that even if the regulation network is a DAG, the stability of the system is not determined solely by the self-regulation loops. Instead, we find that if we draw DAGs randomly (constructed by adding a connection only if the resultant network is still acyclic, Fig. 2c), even if there are no self interactions, the largest eigenvalue still scales approximately with $\sqrt{N}$, suggesting that it is still possible for such a network to go unstable. Nevertheless, there is a negative offset in ${\lambda }_{M,{r}_{\max }}$ compared to the fully random case (Fig. 2a), implying that the lack of loops does help to stabilize the system.

#### Bipartite structure can maintain stability of large networks

A commonly found motif in the *Escherichia coli* transcription network is the dense-DORs which consist of a set of regulators that combinatorially control a set of output genes^1,29,30^. There are several of these DORs in *E. coli*, each with hundreds of output genes, and they appear to occur in a single layer, i.e., there is no DOR at the output of another DOR. Such a structure can be thought of as a bipartite graph in which there are two types of nodes representing TFs and non-transcription factors (non-TFs), and every directed edge go from a TF to a non-TF. Since such graphs do not contain any regulatory loops (and are therefore also DAGs), we expect them to be more stable than random networks. However, they are a specific subset of DAGs in which none of the TFs are themselves regulated. This is also a key difference between these networks and bipartite, mutualistic networks commonly studied in ecological models^9,10^. In this subsection, we investigate the stability of such networks.

To study this problem, we first group proteins into two categories: *q* TFs and *N*−*q* non-TFs, such that for any general network the components of the Jacobian matrix have the following structure:

17 $${{\bf{M}}}_{{\bf{1}}}=\left(\begin{array}{cc}{{\bf{T}}}_{{\bf{1}}}&{\bf{0}}\\ {{\bf{R}}}_{{\bf{1}}}&{\bf{0}}\end{array}\right)$$

18 $${{\bf{M}}}_{{\bf{2}}}=\left(\begin{array}{cc}{{\bf{T}}}_{{\bf{2}}}&{\bf{0}}\\ {{\bf{R}}}_{{\bf{2}}}&{\bf{0}}\end{array}\right),$$

where **T**_**1**_ (**T**_**2**_) is a *q* × *q* matrix representing the direct (indirect) effect of TFs on TFs while **R**_**1**_ (**R**_**2**_) is a (*N* − *q*) × *q* matrix representing the direct (indirect) effect of TFs on non-TFs, with their elements defined previously (Eqs. (13)–(15)). The non-zero eigenvalues of **M** are therefore the eigenvalues of the sub-matrix **Q** with elements:

19 $${Q}_{ij}={c}_{i}^{ss}({T}_{1,ij}-{T}_{2,ij}).$$

When the network is sparse, each TF only regulates a small fraction of the total number of genes. Since *c*^*ss*^ ~ 1/*N*, the strength of indirect interactions are therefore typically much weaker than that of direct interactions (i.e., the non-zero elements of **M**_**2**_ are much smaller in magnitude than that of **M**_**1**_, Eqs. (14), (15)).

**Fig. 3:**
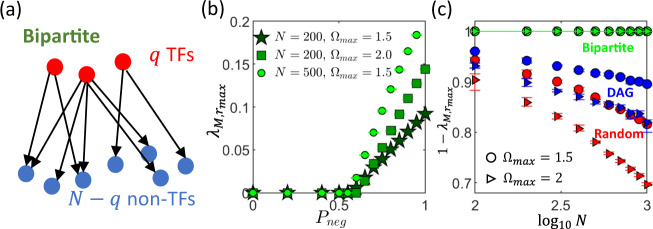
Stability of bipartite networks. **a** When constructing a bipartite interaction network, we group the proteins into transcription factors (TFs, red circles) and non-TFs (blue circles), and only allow directed regulatory interactions to go from a TF to a non-TF. **b** For bipartite networks, there is a critical value for the fraction of inhibitory interactions *P*_neg_ (that is slightly > 0.5) below which the maximal real part of the eigenvalues of the interaction matrix ${\lambda }_{M,{r}_{\max }}=0$ and above which ${\lambda }_{M,{r}_{\max }} \, > \, 0$. In the regime where ${\lambda }_{M,{r}_{\max }}=0$ (which can be considered to be deeply stable since it is far from the point ${\lambda }_{M,{r}_{\max }}=1$ where the system becomes unstable), this value of ${\lambda }_{M,{r}_{\max }}$ stays the same even when the number of different proteins *N* (star markers vs. circles) or interaction strengths ${{{\Omega }}}_{\max }$ (star markers vs. squares) are increased. **c** When there is an equal fraction of up/downregulatory interactions *P*_*neg*_ = 0.5, ${\lambda }_{M,{r}_{\max }}$ is independent of both *N* and ${{{\Omega }}}_{\max }$ for bipartite networks (green markers). This is in contrast to fully random networks (‘Random’, red markers) and random directed acyclic graphs (‘DAG’, blue markers) where the system approaches the instability limit (${\lambda }_{M,{r}_{\max }}=1$) as *N* or ${{{\Omega }}}_{\max }$ (circles to triangles) is increased. This implies that a bipartite network structure can maintain and enhance the stability of the system as *N* or ${{{\Omega }}}_{\max }$ is increased. In both (**b**) and (**c**), each data point is obtained from an average of 10 randomly drawn networks, with error bars indicating the interquartile range. [Other parameters: *h* = 1, *ρ* = 0.01 for fully random and random DAGs, number of TFs for bipartite networks *q* = 0.1*N*].

When constructing random bipartite networks, we only allow TFs to regulate non-TFs (Fig. 3a), implying that **T**_**1**_ = **0**. The matrix **Q** therefore only consists of weak indirect interactions, and we expect the maximal eigenvalue to be smaller than that of random networks and DAGs. Moreover, since in this case **Q** is of rank-1, it has a unique real eigenvalue *λ*_*Q*,*b*_ which can be shown to be (see Supplementary Note 6):

20 $${\lambda }_{Q,b}=-\sum_{i = 1}^{q}{c}_{i}\frac{\partial \mathrm{log}\,{g}_{T}}{\partial {c}_{i}},$$

where $\frac{\partial \mathrm{log}\,{g}_{T}}{\partial {c}_{i}}=\mathop{\sum }\nolimits_{j = 1}^{N}{c}_{j}\frac{\partial \mathrm{log}\,{g}_{j}}{\partial {c}_{i}}$ as defined in Eq. (15) are the elements of the **M**_**2**_ matrix (and therefore small when the interaction density is low). The maximum eigenvalue of the interaction matrix **M** is then given by ${\lambda }_{M,b}=\max ({\lambda }_{Q,b},0)$, since 0 is also an eigenvalue of **M** (see Eqs. (17, 18)).

This expression (Eq. (20)) implies that unlike for fully random networks and random DAGs, the stability of bipartite networks can depend strongly on the ratio of up- and downregulating interactions (see Supplementary Note 4). In particular, there is a limit on the total strength of down-regulation (relative to that of up-regulation) for the system to be stable. For example, if the majority of the interactions are upregulating, *λ*_*Q*,*b*_ should be negative and hence *λ*_*M*,*b*_ must be 0. On the other hand, *λ*_*M*,*b*_ must be positive when the fraction of downregulations is sufficiently high. This tendency for inhibitory (activating) interactions to destabilize (stabilize) the system comes from the indirect effect that a regulator has on itself: a slight increase in the concentration of an inhibitor from its steady-state value will reduce the gene copy number and hence mRNA levels of the regulated gene. The mRNAs of the inhibitor therefore make up a larger fraction of the total mRNA in the cell. Since all mRNAs compete for the shared pool of ribosomes, this in turn causes the inhibitor concentrations to increase further. This positive feedback also exists in the other phases, although its physical origin may be different (see Supplementary Note 4, Supplementary Fig. 2b).

Indeed, by numerically constructing multiple instances of a bipartite network and varying the fraction of inhibitory interactions *P*_neg_, we find that *λ*_*M*,*b*_ = 0 when *P*_neg_ is below a critical value that is approximately (but slightly greater than) 0.5 (Fig. 3b). Importantly, within this regime, the value of *λ*_*M*,*b*_ = 0 is independent of both *N* and the strength of interactions ${{{\Omega }}}_{\max }$ (Fig. 3b, c). This suggests that such a bipartite network structure can help to maintain and enhance the stability of the system, especially for large *N* and ${{{\Omega }}}_{\max }$.

#### Scrambling the interactions of E. coli transcriptional regulatory network can destabilize the system

Real transcription networks, however, are not strictly bipartite graphs—there are autoregulatory elements as well as TFs that regulate other TFs. To investigate how relevant network stability is to biological networks, we obtained the *E. coli* transcriptional regulatory network from ref. ^31^. The network consists of *u* = 5654 regulatory interactions (of which *u*_*p*_ = 3187 are upregulating), with *q* = 211 TFs regulating *N* = 2274 genes. We compared its stability with that of randomly constructed networks with the same *N*, density of interactions $\rho =\frac{u}{{N}^{2}}\approx 0.0011$, and ratio of positive (activating) to negative (inhibitory) regulation.

We first explored two different ways of scrambling the original network: (1) randomly choosing *u* directed connections out of the *N*(*N* − 1) possible connections, and (2) fixing the number of TFs *q* and randomly choosing *u* directed connections out of *qN* possibilities. The second method of scrambling is motivated by the fact that *q* ≪ *N* and the stability of the system is governed solely by the *q* × *q* matrix **Q** representing how TFs affect TFs (Eq. (19)). For each drawn interaction network, we randomly choose *u*_*p*_ of the interactions to be upregulating (*γ*_*ij*_ > 0) and the rest to be downregulating (*γ*_*ij*_ < 0). We draw the fold-change Ω_*ij*_ of each regulatory interaction from a uniform distribution between 1 and ${{{\Omega }}}_{\max }=1000$. This choice of ${{{\Omega }}}_{\max }$ is motivated by the fact that TFs have been shown experimentally to change target protein levels by 100–1000 fold^13^.

**Fig. 4:**
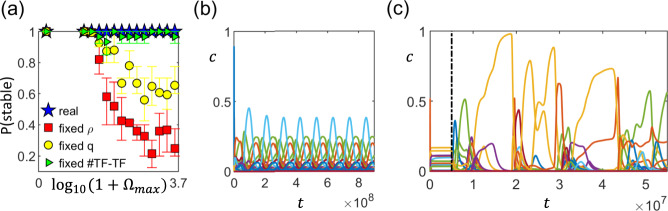
Comparing the *E. coli* transcriptional regulatory network with random networks of the same density. **a** The actual *E. coli* network does not become unstable even when the maximum regulation strength ${{{\Omega }}}_{\max }$ is increased (blue stars). In contrast, as ${{{\Omega }}}_{\max }$ increases, the probability P(stable) of the system having a stable fixed point decreases for scrambled networks of the same interaction density *ρ* = 0.0011, regardless of whether the number of transcription factors (TFs) *q* = 211 is kept fixed (yellow circles) or not (red squares). However, scrambling the network while maintaining the same number of TF-other TF, TF-nonTF, and self interactions can significantly enhance the probability of the system is stable (green triangles). Each of the data points represents an average over 15 sets of 10 regulatory networks, with error bars indicating the interquartile range. [Other parameters: *h* = 2]. **b** A typical example of oscillatory dynamics in protein concentrations *c* when the system no longer has a stable fixed point. [Parameters: ${{{\Omega }}}_{\max }=1585$, *h* = 2]. **c** An example of the system going unstable and exhibiting chaotic behavior when the real network is scrambled at time *t* = 5 × 10^6^ marked by the dashed vertical line. [Parameters: ${{{\Omega }}}_{\max }=1000$, *h* = 5]. In both (**b**) and (**c**), time *t* is in units of 1/*k*_*p*_.

We find that with the real network, the system always converges to a stable fixed-point regardless of the regulation strengths (Fig. 4a). In contrast, for the randomly constructed networks (both with and without keeping *q* fixed), the probability of the system becoming unstable drastically increases when the interactions become too strong (Fig. 4a). This loss of a stable fixed point can give rise to either an oscillatory (Fig. 4b) or chaotic behavior (Fig. 4c). This suggests that for typical regulation strengths and density, the interaction network cannot be random, and that certain structural features of real networks are important for stability.

#### Network stability depends on the density of TF–TF interactions

Since it is the maximal eigenvalue of the *q* × *q* sub-matrix **Q** (Eq. (19)) that determines the stability of the system, and direct regulatory interactions are typically stronger than the indirect background effects, we expect a higher density of direct interactions among TFs to destabilize the system. This suggests that what matters for stability is not only the number of TFs and the total number of regulatory interactions, but also the fraction of those interactions that target TFs.

**Fig. 5:**
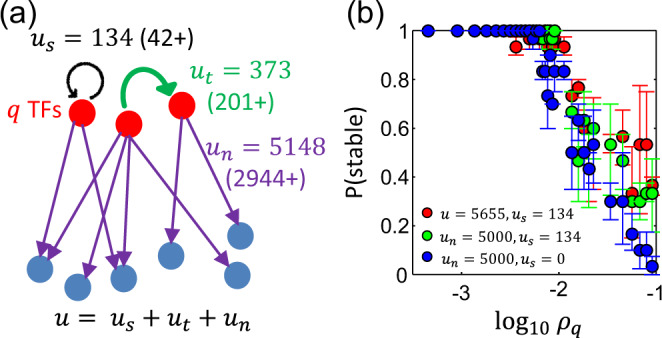
Effect of density *ρ*_*q*_ of transcription factor (TF)-otherTF interactions on stability. **a** In the real network analyzed, there are *u*_*s*_ = 134 self-regulations (of which 42 of them are activating), *u*_*t*_ = 373 TF-other TF interactions (of which 201 of them are activating), and *u*_*n*_ = 5148 TF-nonTF interactions (of which 2944 of them are activating). The total number of interactions is given by *u*. **b** A randomly constructed network is almost always stable when *ρ*_*q*_ is sufficiently low. Above a threshold value, the probability of being stable (P(stable)) decreases with *ρ*_*q*_. This is true with (red and green circles) or without (blue circles) self-interactions, and regardless of whether it is the total number of interactions *u* (red circles) or the number of TF-nonTF interactions *u*_*n*_ (green and blue circles) that is kept constant. Each data point is an average over 15 sets of 10 regulatory networks, with error bars indicating the interquartile range. [Parameters: *N* = 2274, *q* = 211, *h* = 2, ${{{\Omega }}}_{\max }=1000$.].

We therefore analyzed the composition of regulatory interactions in the *E. coli* transcription network, and found that there are (i) *u*_*s*_ = 134 self-regulations (of which 42 are activating), (ii) *u*_*t*_ = 373 TF-other TF interactions (of which 201 are activating), and (iii) *u*_*n*_ = *u* − *u*_*s*_ − *u*_*t*_ = 5148 TF-nonTF interactions (of which 2944 are activating) (Fig. 5a). In comparison, the scrambling method that maintained both the number of TFs and the total number of interactions gives a smaller number of self-interactions (〈*u*_*s*_〉 = 2.5) and a larger number of direct TF-other TF interactions (〈*u*_*t*_〉 = 522) than in the real network.

To investigate if this could be the origin of the enhanced stability of the *E. coli* regulatory network, we tried another scrambling method with the composition of the interactions kept fixed. In particular, after setting the first *q* = 211 (out of *N* = 2274) proteins to be TFs, we randomly draw the numbers of interaction pairs within the three categories (self, TF-otherTF, and TF-nonTF) by choosing each TF and its target separately. The sign of the interactions are then randomly assigned while maintaining the fraction of positive/negative interactions within each of these categories. We find that this scrambling procedure, which fixes the composition of regulatory interactions (in addition to *N*, *q,* and *ρ*), significantly increases the probability of the network having a stable fixed point (Fig. 4a).

Direct interactions among TFs can either be auto-regulatory loops or TFs regulating other TFs. We explored the effects of both of these factors, and found that assuming up- and downregulations to be equally likely, a random network is almost always stable when the density of TF-other TF interactions ${\rho }_{q}=\frac{{u}_{t}}{q(q-1)}$ is sufficiently low (Fig. 5b). Above this threshold value of *ρ*_*q*_, the probability of the system not exhibiting a stable steady-state increases with *ρ*_*q*_ (Fig. 5b). This effect is observed regardless of the number of self-interactions or whether *u*_*n*_ is kept fixed (Fig. 5b).

While this implies that systems with a small number of TF–TF interactions are almost always stable, it does not mean that having a high density of TF–TF interactions will necessarily lead to an unstable system. This can be seen from the fact the probability of the system is stable does not drop sharply with *ρ*_*q*_ (Fig. 5b)—there are still systems with a relatively high density of TF–TF interactions that are still stable. This suggests that in the high *ρ*_*q*_ regime, the details of the interactions become important. For such a network with a large number of TF–TF interactions to be stable, the type and strength of those interactions will need to be more fine-tuned.

The phenomenon that a small *ρ*_*q*_ promotes stability is consistent with the stability of bipartite networks (*ρ*_*q*_ = 0) and the fact that direct regulatory interactions are typically much stronger than the indirect background interactions. Nevertheless, since **Q** (which has contributions from both **T**_**1**_ and **T**_**2**_, Eq. (19)) is not a sparse matrix even when *ρ*_*q*_ is small, we do not expect the maximal eigenvalue ${\lambda }_{M,{r}_{\max }}$ to scale with *ρ*_*q*_ the way it does for a *q* × *q* random matrix with density *ρ*_*q*_. Indeed, we find numerically that the presence of **T**_**2**_ can affect ${\lambda }_{M,{r}_{\max }}$ (Supplementary Note 7, Supplementary Fig. 4), suggesting that the indirect coupling between proteins can also play a role in influencing the stability of the system.

### Effect of degradation rates on protein level stability

So far, we have been working in the limit of fast mRNA degradation, where the stability of the system is governed only by the interaction matrix **M** (Eq. (12)). In this regime, since **M** is independent of degradation rates 1/*τ*_*m*_ and 1/*τ*_*p*_ (see Eqs. (12, 6, 3)), these do not affect whether the system is stable. The relaxation rates are also independent of *τ*_*m*_ and *τ*_*p*_, with the relaxation rate in the absence of interactions given by (from Eq. (11)):

21 $${\beta }_{0}={k}_{p}{c}_{r}^{ss}.$$

However, it is not clear if this insensitivity (of both stability and relaxation rates) to *τ*_*m*_ and *τ*_*p*_ still holds outside of the *τ*_*m*_ ≪ *τ*_*p*_ regime. Within the framework of RMT, a more negative self-regulation term typically increases the relaxation rate and hence has a stabilizing effect^2^. Here, we ask if this is the case by investigating how mRNA and protein degradation rates affect the stability of the system and its relaxation timescale. In particular, can faster mRNA degradation rates help to stabilize a system that would otherwise be unstable if mRNAs degrade too slowly?

#### Values of mRNA and protein degradation rates do not affect whether the system is stable

To investigate how the degradation rates of proteins and mRNAs affect the stability of the system when *τ*_*m*_ is not too small, here we consider the full set of 2*N* equations (Eqs. (9, 10)) and study how the eigenvalues of the (2*N* × 2*N*) Jacobian matrix **J** varies with *τ*_*m*_ and *τ*_*p*_.

To compare the relaxation rates of the full system with the protein relaxation rates when there are no interactions, we work with the transformed Jacobian matrix:

22 $$\tilde{{\bf{J}}}=\frac{1}{{\beta }_{0}}{\bf{J}}.$$

For an arbitrary regulatory network with a corresponding interaction matrix **M** (Eq. (12)), we find that the eigenvalues $\tilde{\lambda }$ of $\tilde{{\bf{J}}}$ are given by (see Supplementary Note 3):

23 $$\tilde{\lambda }=\frac{1}{2}\left(-\omega \pm \sqrt{{\omega }^{2}+4{\lambda }_{M}(1+\omega )}\right)-1,$$

where *λ*_*M*_ are the eigenvalues of **M** as before, and *ω* is a dimensionless quantity given by:

24 $$\omega =\frac{1}{\tau {\beta }_{0}},$$

which reflects the difference between mRNA and protein degradation rates $\left(\frac{1}{\tau }=\frac{1}{{\tau }_{m}}-\frac{1}{{\tau }_{p}}\right)$.

**Fig. 6:**
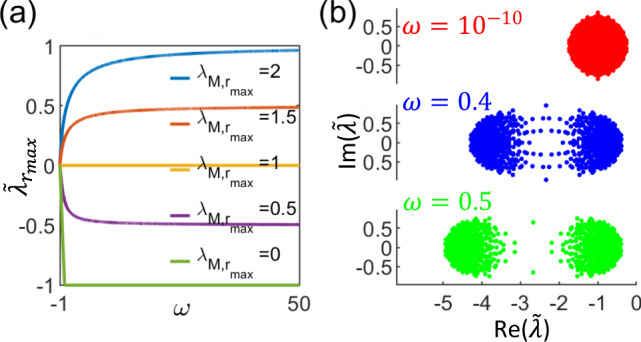
Effect of degradation rates on stability. **a** The system is stable if and only if the maximal real part of the eigenvalues of the interaction matrix ${\lambda }_{M,{r}_{\max }}\le 1$, regardless of the value of *ω* which increases with mRNA degradation rates (Eq. (24)). The scaled eigenvalues $\tilde{\lambda }\to {\lambda }_{M}-1$ in the limit of fast mRNA degradation rate *ω* → *∞* (Eq. (23)). **b** Eigenvalue spectrum for different degradation rates *τ*. When mRNA and protein degradation rates are comparable, all eigenvalues fall within a circular region (red). On the other hand, when *τ*_*m*_ ≪ *τ*_*p*_, the eigenvalue spectrum approximately resembles two circular regions, one corresponding to the dynamics of mRNAs and one for that of proteins. In this limit, increasing mRNA degradation rate only shifts the eigenvalues for the mRNA sector to more negative values, leaving the maximal real part of the eigenvalues approximately unchanged, *ω* = 0.5 (green) vs *ω* = 0.4 (blue).

Since on average cell volume increases exponentially with rate (see Eq. (8)):

25 $$\mu ={k}_{p}{\phi }_{r}-\frac{1}{{\tau }_{p}},$$

a growing cell has to satisfy the condition $\frac{1}{{\tau }_{p}{k}_{p}{\phi }_{r}} \, < \, 1$. Therefore, since *τ*_*m* _≥ 0, we have *ω* ≥ −1. The expression for $\tilde{\lambda }$ (Eq. (23)) therefore implies that the system is stable if and only if ${\lambda }_{M,{r}_{\max }}\le 1$, regardless of the value of *τ*_*m*_ and *τ*_*p*_ (Fig. 6a). We find that despite differences in the details of the model, this conclusion still holds in the other phases (see Supplementary Note 3).

Therefore, unlike what has been argued in the literature and what one might expect from RMT, changing mRNA nor protein degradation rates has no effect on whether the overall system is stable. If steady-state protein concentrations are unstable because ${\lambda }_{M,{r}_{\max }}$ is too large (e.g., when interactions are too strong), increasing mRNA or protein degradation rates can never help to stabilize the system.

Importantly, this finding also implies that our results for how structural features of the transcription network affects stability holds outside the regime of fast mRNA degradation, since stability only depends on **M**.

#### Increasing mRNA degradation rate can improve response times, but only up to some limit

Besides system stability, another quantity of biological interest is the response time of the system to perturbations, which is especially relevant for cells experiencing changes in nutrient conditions^32,33^. Since this relaxation timescale is determined by the slowest eigenvalue of the Jacobian matrix, here we discuss how the maximal real part of the eigenvalues ${\tilde{\lambda }}_{{r}_{\max }}$ changes with *τ*.

The expression for $\tilde{\lambda }$ (Eq. (23)) implies that when the system is stable (${\lambda }_{M,{r}_{\max }}<1$), the rate at which the system relaxes to steady-state initially increases as *ω* increases from −1, but eventually plateau off − in the *ω* → *∞* limit (where *τ*_*m*_ ≪ *τ*_*p*_), $\tilde{\lambda }\to {\lambda }_{M}-1$ (Eq. (23), Fig. 6a). This implies that there is some benefit to having fast mRNA degradation in terms of response times, but once mRNA degrades much faster than proteins, further increasing mRNA degradation rate no longer affects the response time of the system. The eigenvalue spectrum in this *τ*_*m*_ ≪ *τ*_*p*_ limit appears to consist of two circular regions, one for the dynamics of mRNAs and the other for that of proteins (Fig. 6b), reminiscent of the RMT’s circular law. Increasing *τ*_*m*_ only shifts the eigenvalues corresponding to the mRNA sector and hence does not affect ${\tilde{\lambda }}_{{r}_{\max }}$. This is consistent with the fact that when *τ*_*m*_ ≪ *τ*_*p*_, the dynamics of the overall system is governed only by the protein sector (Eq. (11)). Therefore, the slowest relaxation rate back to steady-state levels depends only on **M** and increasing mRNA degradation rate no longer improves the response time.

## Discussion

In systems with a large number of interacting components, the question of stability is often an important one, as results from RMT predict instability when the system size *N* becomes too large or interactions become too strong. In the context of gene expression, transcriptional regulation is crucial for cells to adapt to different environmental conditions by changing their gene expression levels. It is therefore important for transcriptional regulatory networks (TRNs) to be able to accommodate a large number of regulatory interactions without the system going unstable. However, we find here that similar to the intuition provided by RMT, $\lambda \sim \sqrt{N}$ for a fully random regulation network, suggesting that the system will go unstable as the number of genes exceeds a threshold. In fact, based on typical values for the density of actual regulatory networks and interaction strengths, we find that the system has a high probability of being unstable if the TRN is randomly constructed.

Besides the number of genes, and the density and strengths of interactions, there are other factors that can affect the stability of the system, one of which is the network topology. This aspect is particularly relevant in this system since TRNs are far from being random but instead consist of recurring motifs. While the properties of these specific motifs have been widely studied and shown to be important for specific functions such as adaptation, robustness, and fast response to environmental changes^1,29,30^, how they contribute to the overall stability of the network is less clear. We find here that global structural features of the network, which are fundamentally shaped by many of these motifs, can play a huge role in determining the stability of the system. In particular, given the same number of proteins, TFs, interaction density, and regulation strengths, a network that resembles a bipartite graph with a lower density of TF-otherTF interactions *ρ*_*q*_ has a higher chance of being stable. The significance of *ρ*_*q*_ fundamentally arises because of two main factors: (i) the eigenvalues of the Jacobian matrix and hence the stability of the system about its steady-state are governed only by the TF sector (i.e., how perturbations in TF concentrations affect TFs), and (ii) for a sparse regulatory network, the indirect background interactions arising from competition for ribosomes between different genes are typically much weaker than the direct regulatory interactions.

TRNs are also known to be scale-free, having a power-law out-degree distribution. This is consistent with the fact that most TFs only regulate a small number of genes, but there are TFs (often referred to as master regulators) that regulate a very large number of genes. Within a more abstract model of gene regulatory dynamics, the presence of these outgoing hubs has been shown to significantly increase the probability of the system reaching a stable target phenotype when the interaction strengths are allowed to vary while the network topology is kept fixed^34^. Here, we find that having a low *ρ*_*q*_ can already significantly stabilize the system without the need to control the degree distributions. Nevertheless, having just a few master regulators may contribute to the network having a low *ρ*_*q*_ if for instance most of the regulations on TFs are carried out by the master regulators (and non-master regulators predominantly regulate non-TFs).

Besides the structural features of the network, another factor that could affect stability is the degradation rates of mRNA and proteins. Based on RMT, one may expect faster degradation to stabilize the system. This has in fact been argued to be the case^5,6^. However, by taking into account the dynamics of protein concentrations and how it couples to the dynamics of mRNA levels, we find that this is not the case. Instead, the stability of the system depends solely on the regulatory network and the strengths of those regulations—if the system is unstable, it will be unstable regardless of how fast mRNA or protein degrades. This highlights the importance of taking into account key aspects of the interactions (through the form of the dynamical equations) when analyzing the stability of large coupled systems, similar in spirit to studies of ecological models where explicitly considering interactions mediated through competition for nutrients can give drastically different results from assuming random pairwise interactions between species^7^. This prediction can also potentially be tested in the lab by varying the degradation rates of mRNAs (e.g., by using genetically modified RNases) or proteins (e.g., by using genetically modified proteases) in the cell and observing the dynamics of protein concentrations.

From an evolutionary perspective, there are many possible factors (such as the range of gene expression levels, environmental conditions, response time^32,33^, level of unwanted crosstalk^35^, etc.) that drive the addition or removal of regulatory connections. Our findings suggest that in addition to these considerations, another fundamental factor is the stability of the overall network. For example, there could be many ways of achieving a certain task such as allowing the cell to switch between two desired gene expression levels in two different nutrient conditions, but the only ones that can survive are those that also maintain the stability of the system. In other words, the stability of the system may have played a role in shaping current existing regulatory networks through the evolutionary process. Our approach can therefore provide insights into the design and evolutionary constraints for a functional regulatory network, which may potentially be useful for guiding the construction of synthetic genetic circuits^36–38^. In the future, the ability to experimentally engineer a large, random regulatory circuit within cells could also allow testing of the results we have described.

In addition to transcriptional regulation, gene expression is also regulated at the post-transcriptional (e.g., through small-RNAs or micro-RNAs) and post-translational (e.g., through post-translational modifications) level. Our framework can be extended to take into account these effects (see Supplementary Note 8 for an example). How the stability of the system is affected by the coupling between these different forms of regulation with potentially different network structures is an interesting question that we leave for future work. Besides stability (determined by the eigenvalues of **J**), in the future, it could also be instructive to investigate the spread of perturbations within the regulatory network (i.e., the eigenvectors of **J**). This is analogous to the study of how concentration perturbations propagate in protein–protein interaction networks within the cell^39^.

### Reporting summary

Further information on research design is available in the Nature Research Reporting Summary linked to this article.

## Supplementary information

Supplementary Information

Reporting Summary

## Data Availability

The *E. coli*. transcriptional regulatory network data that support the findings of this study is available in the supplementary files of the paper (ref. ^31^): 10.1073/pnas.1702581114. This data used for analysis is also available in a MATLAB data file on GitHub repository^40^: https://github.com/yipeiguo/TRNstability.

## Code availability

All simulations and data analysis are carried out using codes written in MATLAB R2019a. These can be found on GitHub repository^40^: https://github.com/yipeiguo/TRNstability.
