# Publisher Correction: Exploring the effect of network topology, mRNA and protein dynamics on gene regulatory network stability

**DOI:** 10.1038/s41467-021-21415-w

**Published:** 2021-02-08

**Authors:** Yipei Guo, Ariel Amir

**Affiliations:** 1 John A. Paulson School of Engineering and Applied Sciences, Harvard University, Cambridge, MA USA; 2 Program in Biophysics, Harvard University, Boston, MA 02115 USA

**Keywords:** Dynamical systems, Regulatory networks, Complex networks

Correction to: *Nature Communications* 10.1038/s41467-020-20472-x, published online 8 January 2021.

The original version of this Article contained errors in Equations 17 and 18. Both equations incorrectly read: M_1_ = (T_1_0R_1_0) and M_2_ = (T_2_0R_2_0), respectively. The correct form of Equations 17 and 18 are: $$M_1 = \left( {\begin{array}{*{20}{c}} {T_1} & 0 \\ {R_1} & 0 \end{array}} \right)$$ and $$M_2 = \left( {\begin{array}{*{20}{c}} {T_2} & 0 \\ {R_2} & 0 \end{array}} \right)$$, respectively. The original version of this Article also contained an error in the fifth paragraph of the Results subsection. The Model, immediately prior to equation 8, which incorrectly read ‘numbers _*pi*_ for *i* = 1, ..., *N*.’ The correct version should read ‘numbers *p*_*i*_ for *i* = 1, ..., *N*’. Furthermore, an error occurred in the seventh paragraph of the Results subsection The Model, immediately prior to equation 9,  which incorrectly read ‘$V = \mathop {\sum}{{_i{_p{_i}}}}$’. The correct version should read ‘$V = \mathop {\sum }{_i} p_i$’. These have been corrected in both the PDF and HTML versions of the Article.

