# Supplementary material for: Exploring the effect of network topology, mRNA and protein dynamics on gene regulatory network stability
Source: Nat Commun. 2021 Jan 8;12:130. doi: 10.1038/s41467-020-20472-x (PMC7794440; doi:10.1038/s41467-020-20472-x)
Supplement: Supplementary file 1 — Supplementary Information [file 41467_2020_20472_MOESM1_ESM.pdf]

Exploring the effect of network topology, mRNA and protein dynamics on gene regulatory network stability (Supplementary Information)

Parameter	Definition/Description	How value is set in simulations
Transcription and transcriptional regulation		
k_m	transcription rate of a single RNA polymerase	N/A (fast mRNA degradation limit)
τ_m	mRNA lifetime	N/A (fast mRNA degradation limit)
g_i	effective gene copy number of i	$g_i(\vec{\mathbf{c}}) = g_{i0} \prod_j (1 + \gamma_{ij} f_{ij}(c_j))$ (Eqn. 3)
g_{i0}	effective gene copy number of i if it were unregulated	drawn from a uniform distribution between 0 and 1
ϕ_i	gene allocation fraction of i	$\frac{g_i}{\sum_k g_k}$ (Eqn. 6)
ϕ_{i0}	gene allocation fraction of i without any regulatory interactions	$\frac{g_{i0}}{\sum_k g_{k0}}$
γ_{ij}	controls the type and strength of regulation	$\gamma_{ij} = \begin{cases} \Omega_{ij} - 1 & \text{if } \gamma_{ij} > 0 \\ \frac{1}{\Omega_{ij}} - 1 & \text{if } \gamma_{ij} < 0 \end{cases}$ (Eqn. 4)
Ω_{ij}	fold-change of each regulatory interaction, controls strength of interaction	drawn from a distribution $P(\Omega)$ (either uniform or log-uniform) between 1 and Ω_{max}
f_{ij}	How protein j affects gene copy number of i	$f_{ij}(c_j) = \frac{c_j^h}{K_{ij}^h + c_j^h}$ (Eqn. 5)
h	Hill coefficient of f_{ij}	set to be $1, 2$ or 5
K_{ij}	concentration of j at which $f_{ij} = 0.5$	set to be ϕ_{j0}
Translation		
k_p	translation rate of a single ribosome	constant, time is measured in units of $1/k_p$.
τ_p	protein lifetime	N/A, does not affect dynamics of protein concentrations in the limit of fast mRNA degradation.

Supplementary Table 1: List of model parameters, their definitions and how they are chosen in the simulations.

Supplementary Note 1
Example of a thermodynamic model of RNA polymerases and transcription factors binding to DNA

We consider the scenario where a gene has 1 promoter site and L regulatory sites, each corresponding to a binding site for a different transcription factor.

Let $q_i = \frac{c_i}{K_i} = e^{-\beta(\epsilon_i - \mu_i)}$ be the binding affinities of each site $i = 0, 1, ..., L$, where c_i is the concentration of the protein i, K_i and ϵ_i are respectively the dissociation constant and binding energy between protein i and site i, and μ_i is the chemical potential of i. We choose the index $i = 0$ to represent binding of RNA polymerase to the promoter and the other indices represent TF binding to the corresponding regulatory site.

The state of the system is then given by $\vec{\boldsymbol{\sigma}}$ with $\sigma_i = \{0, 1\}$ representing whether the ith binding site is occupied. We allow pairwise interactions between RNAP and each of the TFs, but neglect any pairwise interactions among the TFs, such that the free energy E of any state is given by:

$$E(\vec{\boldsymbol{\sigma}}) = \sum_i (\epsilon_i - \mu_i)\sigma_i - \sum_j \frac{\log w_{0j}}{\beta}\sigma_0\sigma_j, \qquad \text{(Supplementary Equation 1)}$$

where $w_{0j} \geq 0$ captures the strength and nature of the pairwise interaction between a bound RNAP and a bound TF j. Specifically, $w > 1$ indicates a positive interaction (with the TF up-regulating gene expression), $w = 1$ indicates no interaction, while $w < 1$ indicates a repulsive interaction. The limit where $w_{0j} = 0$ corresponds to the case where the TF is a steric inhibitor i.e. binding of j blocks RNAP from binding to the promoter.

Denoting Z^{ON} (Z^{OFF}) as the sum over the weights of all possible RNAP-bound 'ON' (RNAP-unbound 'OFF') configurations, the equilibrium probability P_b of RNAP binding to the promoter is given by

$$\begin{aligned} P_b &= \frac{Z^{ON}}{Z^{ON} + Z^{OFF}} \\ &= \frac{q_0 \prod_{i=1}^{L}(1 + w_{0i}q_i)}{\prod_{i=1}^{L}(1 + q_i) + q_0 \prod_{i=1}^{L}(1 + w_{0i}q_i)} \\ &= P_{b0}F_{reg}(\vec{\mathbf{c}}), \end{aligned} \qquad \text{(Supplementary Equation 2)}$$

where $P_{b0} = \frac{q_0}{1+q_0}$ is the probability of RNA polymerase being bound to the promoter in the absence of any transcriptional regulation ($L = 0$). $F_{reg}(\vec{\mathbf{c}})$ is the regulatory function which captures the effect of TFs on the the binding of RNAP to the promoter, and is given by:

$$\begin{aligned} F_{reg}(\vec{\mathbf{c}}) &= \frac{(1 + q_0)\prod_{i=1}^{L}\left(\frac{1+w_{0i}q_i}{1+q_i}\right)}{1 + q_0\prod_{i=1}^{L}\left(\frac{1+w_{0i}q_i}{1+q_i}\right)} \\ &\approx \prod_{i=1}^{L}\left(1 + \frac{(w_{0i} - 1)q_i}{1 + q_i}\right), \end{aligned} \qquad \text{(Supplementary Equation 3)}$$

with the approximation taken in the limit of low RNAP concentrations $q_0 \ll \prod_{i=1}^{L}\left(\frac{1+q_i}{1+w_{0i}q_i}\right)$ and $q_0 \ll 1$.

This model is therefore an example of how a multiplicative form for $F_{reg}(\vec{\mathbf{c}})$ can arise, and serves as a motivation for our choice of regulatory function for the effective gene copy number (which we assume to be proportional to the probability of RNAP binding to promoter). Even though in this model the Hill coefficient is 1 for the effect of individual TFs, one could imagine higher Hill coefficients if there are cooperative effects in the binding of each TF to its binding site.

Supplementary Note 2
Effect of stochasticity in gene expression and during cell division

To explore the effect of stochasticity in gene expression and binomial sampling of molecules during cell division, we carry out Gillespie simulations. In these simulations, we keep track of the number of mRNA and protein molecules of each gene $i = 1, ..., N$. At every time step, the production rate of each mRNA is given by $\Gamma_{mi} = k_m\phi_i(\vec{\mathbf{c}})$, the production rate of each protein is given by $\Gamma_{pi} = k_p\frac{p_i}{\sum_k p_k}r$, and the

degradation rates of each mRNA and protein are given by $\frac{m_i}{\tau_m}$ and $\frac{p_i}{\tau_p}$ respectively. The next event and the time to the next event are then drawn based on these rates. We assume that the cell divides whenever the volume (i.e. total protein number) reaches a threshold value (2×10^4 in Supplementary Fig. 1). When the protein number is large and the system is stable, we find that protein concentrations fluctuate around the steady-state solution obtained from the corresponding deterministic dynamical equations (Supplementary Fig. 1).

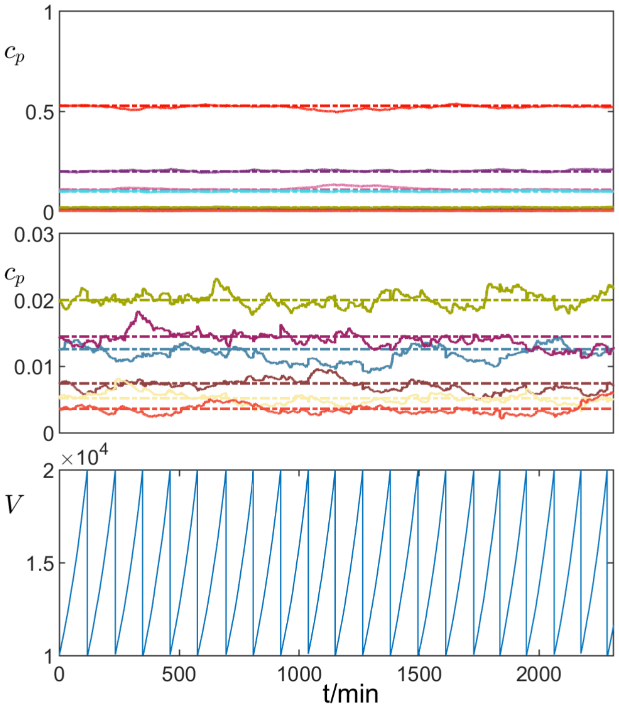

Supplementary Figure 1: Gillespie simulations of gene expression and binomial sampling of mRNA and protein numbers during cell division. The protein concentrations c_p obtained from a single simulation (solid lines in top, middle panels) fluctuate around the steady-state solution obtained from deterministic dynamical equations (dashed horizontal lines, obtained from solving main text Eqns.9, 10). Different colors represent different proteins, the middle panel is a zoomed-in view of the top panel in the low c_p regime. The bottom panel shows the corresponding changes in cell volume V, with vertical lines indicating cell division events, at which point the number of mRNA and protein molecules in the daughter cell is sampled from a binomial distribution. [Parameters: $N = 10$, random interaction network with $\rho = 0.5$, $\Omega_{max} = 20$, $h = 1$, $k_m = 0.3$, $k_p = 0.03$, $\tau_m = 3.5$, $\tau_p = 1 \times 10^8$]

Supplementary Note 3
Dynamics and stability of protein concentrations in different phases

Supplementary Note 3.1
Phase 1: The regime where both RNAPs and ribosomes are limiting ($n < n_c$, $r < r_c$)

When both RNAPs and ribosomes are limiting, the dynamics of mRNA c_{mi} and protein c_i concentrations within the cell are governed by the following equations (Eqns. 9 and 10 of the main text):

$$\frac{dc_{mi}}{dt} = k_m \phi_i(c) c_n - c_{mi} \left(k_p c_r + \frac{1}{\tau} \right) \qquad \text{(Supplementary Equation 4)}$$

$$\frac{dc_i}{dt} = k_p c_r \left(\frac{c_{mi}}{c_{mT}} - c_i \right), \qquad \text{(Supplementary Equation 5)}$$

where k_m and k_p are constants characterizing the transcription and translation rates of a single RNA polymerase and ribosome respectively, $\phi_i(\vec{c}) = \frac{g_i(\vec{c})}{\sum_j g_j(\vec{c})}$ is the gene allocation fraction (with $g_i(\vec{c})$ being the

effective copy number of gene i), $c_{mT} = \sum_i c_{mi}$ and $\frac{1}{\tau} = \frac{1}{\tau_m} - \frac{1}{\tau_p}$, with τ_m and τ_p being the lifetimes of mRNA and proteins respectively.

The corresponding steady-state concentrations are given by:

$$c_{mi,ss} = \frac{k_m c_n^{ss}}{k_p c_r^{ss} + \frac{1}{\tau}} \phi_i(\vec{\mathbf{c}}^{ss}) \quad \text{(Supplementary Equation 6)}$$

$$c_i^{ss} = \phi_i(\vec{\mathbf{c}}^{ss}). \quad \text{(Supplementary Equation 7)}$$

Since by definition $\sum_i \phi_i = 1$, the total steady-state mRNA concentration is

$$c_{mT,ss} = \frac{k_m \phi_n}{k_p \phi_r + \frac{1}{\tau}}. \quad \text{(Supplementary Equation 8)}$$

We denote the total number of genes by N, and choose the index $N-1$ to represent polymerases (the number of which we also denote as n) and the Nth index to represent ribosomes (the number of which we also denote by r). The Jacobian of the full coupled mRNA-protein system is a $2N \times 2N$ matrix $\mathbf{J} = \begin{bmatrix} \mathbf{A} & \mathbf{B} \\ \mathbf{C} & \mathbf{D} \end{bmatrix}$, where $\mathbf{A} = -\left(\frac{1}{\tau} + k_p c_r\right)\mathbf{I}$ is the $N \times N$ matrix representing how mRNA concentrations affect one another, and $\mathbf{D} = -k_p c_r \mathbf{I}$ is the $N \times N$ matrix representing how protein concentrations affect one another. Since c_i^*'s are independent of τ (Supplementary Equation 7), it is convenient to define

$$\tilde{\mathbf{J}} = \frac{1}{k_p c_r}\mathbf{J} \quad \text{(Supplementary Equation 9)}$$

such that the system is stable if and only if the maximal real part of the eigenvalues of $\tilde{\mathbf{J}}$ is less than 0. The elements of $\tilde{\mathbf{J}}$ are then given by $\tilde{\mathbf{J}} = \begin{bmatrix} \tilde{\mathbf{A}} & \tilde{\mathbf{B}} \\ \tilde{\mathbf{C}} & \tilde{\mathbf{D}} \end{bmatrix}$, with $\tilde{\mathbf{A}} = -\left(\frac{1}{\tau k_p c_r} + 1\right)\mathbf{I}$, $\tilde{\mathbf{D}} = -\mathbf{I}$, and

$$\tilde{B}_{ij} = \begin{cases} \frac{k_m \phi_n}{k_p \phi_r}\frac{\partial \phi_i}{\partial c_j}, & \text{for } j = 1, 2, ..., N-2 \\ \frac{k_m \phi_i}{k_p \phi_r}, & \text{for } j = N-1 \\ -\frac{\phi_i}{\phi_r} c_{mT}, & \text{for } j = N \end{cases} \quad \text{(Supplementary Equation 10)}$$

$$\tilde{C}_{ij} = \begin{cases} \frac{1-\phi_i}{c_{mT}}, & \text{for } j = i \\ -\frac{\phi_i}{c_{mT}}, & \text{for } j \neq i \end{cases} \quad \text{(Supplementary Equation 11)}$$

where we have made the assumption that RNAPs and ribosomes cannot act as transcription factors.

Let $\tilde{\lambda}$ be the eigenvalues of $\tilde{\mathbf{J}}$ with corresponding eigenvectors $\vec{v} = \begin{pmatrix} \vec{v_1} \\ \vec{v_2} \end{pmatrix}$. Then $\tilde{\mathbf{A}}\vec{v_1} + \tilde{\mathbf{B}}\vec{v_2} = \tilde{\lambda}\vec{v_1}$ and $\tilde{\mathbf{C}}\vec{v_1} = (\tilde{\lambda}+1)\vec{v_2}$, which gives $\tilde{\mathbf{C}}\tilde{\mathbf{B}}\vec{v_2} = (\tilde{\lambda}+1)\left(\tilde{\lambda} + \frac{1}{\tau k_p c_r} + 1\right)\vec{v_2}$, where the elements of $\tilde{\mathbf{C}}\tilde{\mathbf{B}}$ are given by:

$$\begin{aligned} (\tilde{C}\tilde{B})_{ij} &= \sum_k \tilde{C}_{ik}\tilde{B}_{kj} \\ &= \begin{cases} \frac{k_m \phi_n}{k_p \phi_r c_{mT}}\left(\frac{\partial \phi_i}{\partial c_j} - \phi_i \sum_k \frac{\partial \phi_k}{\partial c_j}\right), & \text{for } j = 1, 2, ..., N-2 \\ \frac{k_m}{k_p c_{mT}}\left(\frac{\phi_i}{\phi_r} - \phi_i \sum_k \frac{\phi_k}{\phi_r}\right), & \text{for } j = N-1 \\ -\frac{\phi_i}{\phi_r} + \phi_i \sum_k \frac{\phi_k}{\phi_r}, & \text{for } j = N \end{cases} \\ &= \begin{cases} \left(1 + \frac{1}{\tau k_p \phi_r}\right)\frac{\partial \phi_i}{\partial c_j}, & \text{for } j = 1, 2, ..., N-2 \\ 0, & \text{for } j = N-1, N \end{cases} \end{aligned} \quad \text{(Supplementary Equation 12)}$$

This therefore provides a relation between each value of $\tilde{\lambda}$ and its corresponding eigenvalue of $\frac{\partial \phi}{\partial c}$, which we denote by λ_M. Since λ_M is independent of τ, we can find how τ affects $\tilde{\lambda}$ for any given λ_M:

$$\tilde{\lambda} = \frac{1}{2}\left(-\omega \pm \sqrt{\omega^2 + 4\lambda_M(1+\omega)}\right) - 1, \quad \text{(Supplementary Equation 13)}$$

where $\omega = \frac{1}{\tau k_p c_r^*}$.

Supplementary Note 3.2
Phase 2: The regime where RNAPs are in excess and ribosomes are limiting ($n \geq n_c$, $r < r_c$)

Whenever it is the gene copy numbers (instead of RNAPs) that are limiting ($n \geq n_c$), the transcription rate is no longer proportional to the number of RNAPs, and hence it is the mRNA numbers m_i rather than their concentrations that are kept at steady-state levels within the cell. We therefore analyze the the dynamics for m_i and c_i which in this case are given by:

$$\frac{dm_i}{dt} = k_m g_i(c) n_s - \frac{m_i}{\tau_m} \quad \text{(Supplementary Equation 14)}$$

$$\frac{dc_i}{dt} = k_p c_r \left(\frac{m_i}{m_T} - c_i \right), \quad \text{(Supplementary Equation 15)}$$

where $m_T = \sum_j m_j$ is the total number of mRNAs, n_s is the maximal number of RNA polymerases a single gene can accommodate, and the other variables are as defined previously.

The corresponding steady-state mRNA and protein levels are:

$$m_i^{ss} = k_m n_s g_i(\vec{\mathbf{c}}^{ss}) \tau_m \quad \text{(Supplementary Equation 16)}$$

$$c_i^{ss} = \frac{m_i^{ss}}{m_T^{ss}} = \phi_i(\vec{\mathbf{c}}^{ss}), \quad \text{(Supplementary Equation 17)}$$

where we note that as before the steady-state protein concentrations are independent of the degradation lifetimes.

Following the same approach as in the previous section, we define the scaled Jacobian matrix (Supplementary Equation 9), where now the elements of $\tilde{\mathbf{J}}$ are given by $\tilde{\mathbf{A}} = \left(-\frac{1}{\tau_m k_p c_r} \right) \mathbf{I}$, $\tilde{\mathbf{D}} = -\mathbf{I}$

$$\tilde{B}_{ij} = \frac{k_m n_s}{k_p c_r} \frac{\partial g_i}{\partial c_j} \quad \text{(Supplementary Equation 18)}$$

$$\tilde{C}_{ij} = \frac{1}{m_T} (\delta_{ij} - c_i^*), \quad \text{(Supplementary Equation 19)}$$

such that

$$\begin{aligned} (\tilde{C}\tilde{B})_{ij} &= \frac{1}{\tau_m k_p c_r} \frac{1}{g_T} \left(\frac{\partial g_i}{\partial c_j} - c_i \sum_k \frac{\partial g_k}{\partial c_j} \right) \\ &= \frac{1}{\tau_m k_p c_r} \frac{\partial \phi_i}{\partial c_j}. \end{aligned} \quad \text{(Supplementary Equation 20)}$$

The eigenvalues $\tilde{\lambda}$ of $\tilde{\mathbf{J}}$ are hence given by

$$\tilde{\lambda} = \frac{1}{2} \left(-\omega_2 \pm \sqrt{\omega_2^2 + 4\lambda_M(\omega_2 + 1)} \right) - 1, \quad \text{(Supplementary Equation 21)}$$

where $\omega_2 = \frac{1}{\tau_m k_p c_r^{ss}} - 1$, and as before λ_M denote the eigenvalues of $\frac{\partial \phi}{\partial c}$, which are independent of τ. This equation is the same as that in phase 1 (Supplementary Equation 13) with ω replaced by ω_2.

Therefore, in both of these phases we get similar dependence of the stability of the system on degradation rates - the system is always stable as long as $\lambda_M < 1$ and unstable if $\lambda_M > 1$, regardless of the values of ω or ω_2.

Supplementary Note 3.3
Phase 3: The regime where both RNAPs and ribosomes are in excess ($n \geq n_c$, $r \geq r_c$)

In this regime, the dynamics of m_i and c_i are given by:

$$\frac{dm_i}{dt} = \tilde{k_m} g_i(\vec{\mathbf{c}}) - \frac{m_i}{\tau_m} \quad \text{(Supplementary Equation 22)}$$

$$\frac{dc_i}{dt} = \tilde{k_p}(m_i - c_i m_T), \quad \text{(Supplementary Equation 23)}$$

where $\tilde{k_m} = k_m n_s$, and $\tilde{k_p} = \frac{k_p r_s}{V}$ is dependent on cell volume which is linearly increasing over time. It is useful to define the growth rate per unit volume μ_3, which is given by:

$$\mu_3 = \frac{k_m k_p r_s n_s \tau_m g_T}{V} - \frac{1}{\tau_p}. \qquad \text{(Supplementary Equation 24)}$$

At steady-state,

$$m_i^{ss} = \tilde{k_m} g_i(\vec{\mathbf{c}}^{ss})\tau_m \qquad \text{(Supplementary Equation 25)}$$

$$c_i^{ss} = \frac{m_i^{ss}}{m_T^{ss}} = \phi_i(\vec{\mathbf{c}}^{ss}), \qquad \text{(Supplementary Equation 26)}$$

and while these are constant over the whole cell cycle, the rate at which the system goes back to steady-state levels after a perturbation depends on its current volume at that point in time.

The Jacobian matrix of this system can again be written as $\mathbf{J} = \begin{bmatrix} \mathbf{A} & \mathbf{B} \\ \mathbf{C} & \mathbf{D} \end{bmatrix}$, where $\mathbf{A} = -\frac{1}{\tau_m}\mathbf{I}$, $\mathbf{D} = -\tilde{k}_1 m_T \mathbf{I}$, $B_{ij} = \tilde{k_m}\frac{\partial g_i}{\partial c_j}$, and $C_{ij} = \tilde{k_p}(\delta_{ij} - c_i)$. Unlike phases 1 and 2, here $\mathbf{D}$ depends on $m_T = \tilde{k_m} g_T \tau_m$ which is a function of τ_m.

The eigenvalues λ of $\mathbf{J}$ can be found from

$$\tilde{\mathbf{C}}\tilde{\mathbf{B}}\vec{v_2} = \left(\lambda + \frac{1}{\tau_m}\right)\left(\lambda + \tilde{k}_1 m_T\right)\vec{v_2}, \qquad \text{(Supplementary Equation 27)}$$

with

$$\begin{aligned} (CB)_{ij} &= \tilde{k}_0\tilde{k}_1\left(\frac{\partial g_i}{\partial c_j} - c_i\sum_k \frac{\partial g_k}{\partial c_j}\right) \\ &= K\frac{\partial \phi_i}{\partial c_j}, \end{aligned} \qquad \text{(Supplementary Equation 28)}$$

where $K = \tilde{k}_0\tilde{k}_1 g_T$. Unlike the other phases, here we choose not to scale $\mathbf{J}$ by the diagonal elements of $\mathbf{D}$ since we are investigating the effect of τ_m on the eigenvalues and $\mathbf{D}$ itself depends on τ_m.

We therefore have

$$\lambda = \frac{-\left(\frac{1}{\tau_m} + K\tau_m\right) \pm \sqrt{\left(\frac{1}{\tau_m} + K\tau_m\right)^2 - 4K(1 - \lambda_M)}}{2}, \qquad \text{(Supplementary Equation 29)}$$

where as before, λ_M are the eigenvalues of the interaction matrix $\mathbf{M} = \frac{\partial \phi}{\partial c}$.

This implies that the system becomes marginally stable ($\lambda \to 0$), for both $\tau_m \to 0$ and $\tau_m \to \infty$, i.e. in both these limits, even if the system is stable, it takes a long time for it to relax back to its steady-state when perturbed. This suggests that there is an intermediate regime of τ_m for which the system responds fast to perturbations away from steady-state. This 'Goldilocks effect' arises because when τ_m is large, the restoring force for mRNA numbers is small, while for small τ_m, the restoring force for protein concentrations is small.

If we were to consider the scaled relaxation rates $\tilde{\lambda} = \frac{\lambda}{b_{0,3}}$, where $b_{0,3} = K\tau_m$ is the relaxation rate for proteins when there are no transcriptional regulation (which is also the growth rate per unit volume μ_3 in the limit $\tau_p \to \infty$), then

$$\tilde{\lambda} = \frac{1}{2}\left(-\omega_3 \pm \sqrt{\omega_3^2 + 4\lambda_M(\omega_3 + 1)}\right) - 1, \qquad \text{(Supplementary Equation 30)}$$

where $\omega_3 = \frac{1}{K\tau_m^2} - 1$. This expression is the same as that in phases 1 and 2 (Supplementary Equation 13, Supplementary Equation 21), with ω and ω_2 now replaced by ω_3. Therefore, as before, the system is always stable as long as $\lambda_M < 1$ and unstable if $\lambda_M > 1$, regardless of the value of ω_3.

Supplementary Note 4
Effect of sign of regulatory interactions on stability

In this section, we investigate how the relative fraction of up- and down- regulatory interactions affect the maximal eigenvalue $\lambda_{M,r_{max}}$ of the interaction matrix.

We find that for random and DAG networks, the fraction of up-regulating interactions p_{up} does not significantly affect $\lambda_{M,r_{max}}$ (Supplementary Fig. 2a). However, for bipartite networks (which do not have any direct interactions between TFs), having only down- regulating interactions ($p_{up} = 0$) increases $\lambda_{M,r_{max}}$ dramatically compared to the scenario of having $p_{up} = 0.5$ (Supplementary Fig. 2b). This is consistent with the tendency for inhibitory (activating) regulations to destabilize (stabilize) the system, which comes from the indirect effect that a regulator has on itself: a slight increase in the concentration of an inhibitor from its steady-state value will reduce the gene copy number and hence mRNA levels of the regulated gene. The mRNAs of the inhibitor therefore make up a larger fraction of the total mRNA in the cell. When ribosomes are limiting (phases 1 and 2), all mRNAs compete for the shared pool of ribosomes, and a higher mRNA fraction therefore causes the inhibitor concentrations to increase further. In phase 3, the reduction in mRNA levels of the regulated gene reduces the rate at which proteins are made. This slowing down of the increase in cell volume causes the inhibitor protein concentration to increase. This effect is much smaller in the case of random and DAG networks because their stability is dominated by the stronger, direct interactions among TFs.

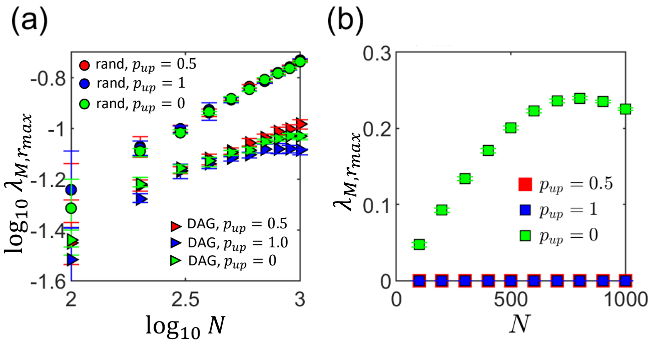

Supplementary Figure 2: Effect of sign of regulatory interactions on maximum real part of the eigenvalues of the interaction matrix $\lambda_{M,r_{max}}$. (a) For fully random ('rand', circular markers) and random directed acyclic graphs ('DAG', triangular markers) regulatory networks, $\lambda_{M,r_{max}}$ is approximately the same when there is an equal fraction of up- and down- regulatory interactions (red markers, '$p_{up} = 0.5$'), when all interactions are up-regulating (blue markers, '$p_{up} = 1$') and when all interactions are down-regulating (green markers, '$p_{up} = 0$'). [Other parameters: $k_p = 1$, $\rho = 0.01$, $h = 1$, $\Omega_{max} = 1.5$.] (b) For bipartite interaction networks, up- regulating interactions are stabilizing while down-regulating interactions are destabilizing. These results hold in all phases of the model (since it is always the same interaction matrix that matters), even though the physical origin of the background, indirect interactions change between the three phases. In both (a) and (b), each data point is obtained from an average of 10 randomly drawn networks, with error bars indicating the interquartile range. [Parameters: $k_p = 1$, $h = 1$, $\Omega_{max} = 1.5$, $\rho = 0.01$ for fully random and random DAGs in (a), number of TFs $q = 0.1N$ for bipartite networks in (b).]

Supplementary Note 5
Effect of distribution of fold-change Ω_{ij} on stability

In this section, we investigate the effect that the distribution of fold-changes Ω_{ij} of the regulatory interactions has on the maximal eigenvalue $\lambda_{M,r_{max}}$ of the interaction matrix.

In the main text, all the simulations were carried out with Ω drawn from a uniform distribution. For any fixed value of Ω_{max}, having $P(\Omega) \sim \frac{1}{\Omega}$ (such that the logarithm of Ω is uniformly distributed [1, 2]) would result in a lower $\langle\Omega\rangle$ and a higher fraction of weaker interactions. Nevertheless, we find that the qualitative behavior of how $\lambda_{M,r_{max}}$ scales with N remains unchanged (Supplementary Fig. 3).

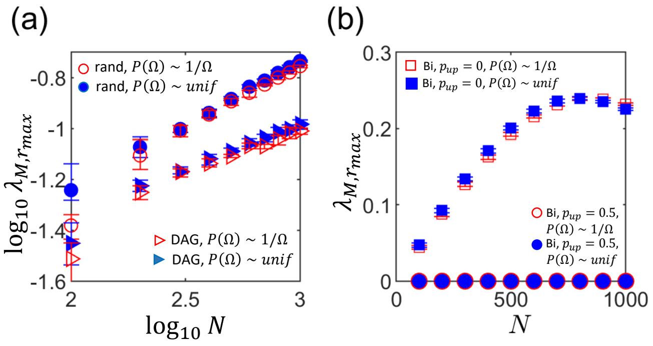

Supplementary Figure 3: Effect of fold change distribution on how the maximal real part of the eigenvalues of the interaction matrix $\lambda_{M,r_{max}}$ varies with the number of different proteins N. (a) For fully random ('rand', circular markers) and random directed acyclic graphs ('DAG', triangular markers) regulatory networks, $\lambda_{M,r_{max}} \sim \sqrt{N}$ both when fold-changes Ω_{ij} are drawn from a uniform distribution (blue markers), and when they are drawn from a $1/\Omega$ distribution (red markers). (b) For bipartite interaction networks ('Bi'), the qualitative behavior of how $\lambda_{M,r_{max}}$ varies with N is also independent of the distribution $P(\Omega)$ of fold-changes regardless of the fraction of up-regulating interactions p_{up}. In both (a) and (b), each data point is obtained from an average of 10 randomly drawn networks, with error bars indicating the interquartile range. [Parameters: $k_p = 1$, $h = 1$, $\Omega_{max} = 1.5$, $\rho = 0.01$ and $p_{up} = 0.5$ for fully random and random DAGs in (a), number of transcription factors $q = 0.1N$ for bipartite networks in (b).]

Supplementary Note 6
Eigenvalue of Jacobian matrix for a bipartite regulatory network

For a bipartite regulatory network, the relevant $q \times q$ sector of the Jacobian matrix $\mathbf{Q}$ (main text Eqn. 19) is given by:

$$\mathbf{Q} = -\vec{\mathbf{c}}\vec{\mathbf{a}}^T, \qquad \text{(Supplementary Equation 31)}$$

where c_i is the concentration of TF i, and $a_i = \frac{\partial log g_T}{\partial c_i}$. Since this is a rank-1 matrix, it only has one eigenvalue λ_b with corresponding eigenvector $\vec{\mathbf{v}}$ such that

$$-c_i \sum_j a_j v_j = \lambda_b v_i, \qquad \text{(Supplementary Equation 32)}$$

for all $i = 1, 2, ..., q$. This implies that

$$-\sum_i a_i c_i \sum_j a_j v_j = \lambda_b \sum_i a_i v_i. \qquad \text{(Supplementary Equation 33)}$$

Therefore

$$\lambda_b = -\sum_i a_i c_i, \qquad \text{(Supplementary Equation 34)}$$

and

$$v_i = c_i. \qquad \text{(Supplementary Equation 35)}$$

Supplementary Note 7
Effect of density of TF-otherTF interactions ρ_q on maximum eigenvalue $\lambda_{M,r_{max}}$

Here, we investigate how the density of TF-otherTF interactions ρ_q affects the maximal eigenvalue $\lambda_{M,r_{max}}$ of the interaction matrix. We find that without any auto-regulation loops, increasing ρ_q increases $\lambda_{M,r_{max}}$, which is consistent with our observation that the probability of the system going unstable increases when ρ_q is too large (Fig. 5b).

These values of $\lambda_{M,r_{max}}$ can be higher than the maximum eigenvalue of the corresponding matrix $\mathbf{Q_1}$ consisting only of the direct interactions i.e. $Q_{1,ij} = c_i \frac{\partial \log g_i}{\partial c_j}$ (Supplementary Fig. 4), especially for small

values of ρ_q, suggesting that the indirect interactions can potentially play a role in affecting the stability of the system. In fact, in the limit where $\rho_q = 0$ (i.e. bipartite network), stability is only determined by these indirect interactions. Nevertheless, these indirect interactions are much weaker than the direct interactions, which accounts for the stability of the system at low ρ_q.

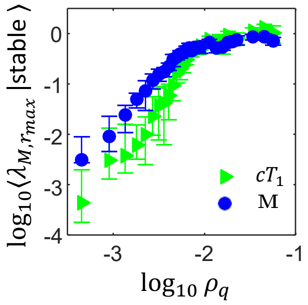

Supplementary Figure 4: Effect of density ρ_q of TF-otherTF interactions on the maximal real part of the eigenvalues of the interaction matrix $\lambda_{M,r_{max}}$ among systems that are stable. When the number of self-interactions $u_s = 0$ and the number of transcription factor (TF)-otherTF interactions $u_n = 5000$, the average $\lambda_{M,r_{max}}$ ('M', blue circles) among stable systems increases with the density ρ_q of TF-otherTF interactions, until reaching a threshold value of ρ_q above which where it stays approximately constant. The maximal real part of the eigenvalues of the corresponding matrix $\mathbf{Q_1}$ consisting only of the direct interactions i.e. $Q_{1,ij} = c_i \frac{\partial \log g_i}{\partial c_j}$ (green triangles) can be lower when ρ_q is small. Each data point is an average over 15 sets of 10 regulatory networks, with error bars indicating the interquartile range. (Other parameters: $N = 2274$, $q = 211$, $k_p = 1$, $h = 2$, $\Omega_{max} = 1000$.)

Supplementary Note 8
Allowing post-translational modifications in the model

Suppose each protein has to undergo some form of modification before they can be functional. In phase 1, the dynamics of the number of mRNAs m_i, proteins p_i, functional proteins p_i^* of each gene i can in general be written as:

$$\frac{dm_i}{dt} = k_m \phi_i(\vec{\mathbf{c}^*}) n^* - \frac{m_i}{\tau_m} \quad \text{(Supplementary Equation 36)}$$

$$\frac{dp_i}{dt} = k_p \frac{m_i}{\sum_j m_j} r^* - \frac{p_i}{\tau_p} - \psi_i(\vec{\mathbf{c}^*}) p_i \quad \text{(Supplementary Equation 37)}$$

$$\frac{dp_i^*}{dt} = \psi_i(\vec{\mathbf{c}^*}) p_i - \frac{p_i^*}{\tau_p}, \quad \text{(Supplementary Equation 38)}$$

where ϕ_i is the gene allocation fraction as defined in our main model (which now depends on concentrations of the functional forms of the transcription factors), and we have assumed that the rate of modification of p_i into p_i^* is proportional to p_i, which is analogous to the reaction being first order in the substrate i. The modification rate also depends on the concentrations of other proteins involved in the modification through the function $\psi_i(\vec{\mathbf{c}^*})$. As an example, for the case of a 1-step enzymatic reaction e.g. phosphorylation of a protein i by a kinase K: $i + K \underset{k_b}{\overset{k_f}{\rightleftharpoons}} i\text{-}K \xrightarrow{k_c} i^* + K$, solving the dynamics of each component (i, i^*, K and i-K) leads to $\psi_i(\vec{\mathbf{c}^*}) = \frac{k_f k_c}{k_b + k_c} c_K^*$, where c_K^* is the concentration of the free kinase, and we have made the approximation that the concentration of the intermediate complex i-K does not change on the time-scale of the formation of product i^* (quasi-steady-state approximation). If we further assume that the total kinase concentration (including both the free and bound forms) is fixed at a constant value of $c_{K,tot}^*$, we recover the familiar Michaelis-Menten kinetics, with $\psi_i(\vec{\mathbf{c}^*}) = \frac{k_c c_{K,tot}^*}{k_m + c_i}$, where c_i is the concentration of i and $k_m = \frac{k_b + k_c}{k_f}$.

Approximating the volume of the cell as $V = \sum_i p_i + p_i^*$, the dynamics of concentrations are then given by:

$$\frac{dc_{mi}}{dt} = k_m \phi_i(\vec{\mathbf{c}^*}) c_n^* - c_{mi} \left(k_p c_r^* + \frac{1}{\tau_m} - \frac{1}{\tau_p} \right) \qquad \text{(Supplementary Equation 39)}$$

$$\frac{dc_i}{dt} = k_p c_r^* \left(\frac{c_{mi}}{\sum_j c_{mj}} - c_i \right) - \psi_i(\vec{\mathbf{c}^*}) c_i \qquad \text{(Supplementary Equation 40)}$$

$$\frac{dc_i^*}{dt} = \psi_i(\vec{\mathbf{c}^*}) c_i - k_p c_r^* c_i^*. \qquad \text{(Supplementary Equation 41)}$$

With such a model, it is then possible to investigate how the coupling of both the transcriptional regulatory network and the network of post-translational modifications would affect stability of the system. We leave this interesting question for future work.

Supplementary References

[1] A. Berger and T. P. Hill, *An introduction to Benford's law*. Princeton University Press, 2015.

[2] R. M. Fewster, "A simple explanation of benford's law," *The American Statistician*, vol. 63, no. 1, pp. 26–32, 2009.
