# Supplementary material for: Exploring the effect of network topology, mRNA and protein dynamics on gene regulatory network stability
Source: Nat Commun. 2021 Jan 8;12:130. doi: 10.1038/s41467-020-20472-x (PMC7794440; doi:10.1038/s41467-020-20472-x)
Supplement: Supplementary file 2 — Reporting Summary [file 41467_2020_20472_MOESM2_ESM.pdf]

nature research

Corresponding author(s): Ariel Amir

Last updated by author(s): Nov 29, 2020

Reporting Summary

Nature Research wishes to improve the reproducibility of the work that we publish. This form provides structure for consistency and transparency in reporting. For further information on Nature Research policies, see our Editorial Policies and the Editorial Policy Checklist.

Statistics

For all statistical analyses, confirm that the following items are present in the figure legend, table legend, main text, or Methods section.

n/a	Confirmed	
☐	☒	The exact sample size (n) for each experimental group/condition, given as a discrete number and unit of measurement
☒	☐	A statement on whether measurements were taken from distinct samples or whether the same sample was measured repeatedly
☒	☐	The statistical test(s) used AND whether they are one- or two-sided<br>*Only common tests should be described solely by name; describe more complex techniques in the Methods section.*
☒	☐	A description of all covariates tested
☒	☐	A description of any assumptions or corrections, such as tests of normality and adjustment for multiple comparisons
☐	☒	A full description of the statistical parameters including central tendency (e.g. means) or other basic estimates (e.g. regression coefficient) AND variation (e.g. standard deviation) or associated estimates of uncertainty (e.g. confidence intervals)
☒	☐	For null hypothesis testing, the test statistic (e.g. F, t, r) with confidence intervals, effect sizes, degrees of freedom and P value noted<br>*Give P values as exact values whenever suitable.*
☒	☐	For Bayesian analysis, information on the choice of priors and Markov chain Monte Carlo settings
☒	☐	For hierarchical and complex designs, identification of the appropriate level for tests and full reporting of outcomes
☒	☐	Estimates of effect sizes (e.g. Cohen's d, Pearson's r), indicating how they were calculated

Our web collection on statistics for biologists contains articles on many of the points above.

Software and code

Policy information about availability of computer code

Data collection	Simulations of the gene expression model were performed using codes written in MATLAB R2019a. These codes are available on GitHub repository (https://github.com/yipeiguo/TRNstability, DOI:10.5281/zenodo.4294914). The actual E.coli transcriptional regulatory network used was obtained from a paper referenced in the text and described in the `Data' section below.
Data analysis	Analysis of the stability of the system was performed using codes written in MATLAB R2019a. These codes are available on GitHub repository (https://github.com/yipeiguo/TRNstability).

For manuscripts utilizing custom algorithms or software that are central to the research but not yet described in published literature, software must be made available to editors and reviewers. We strongly encourage code deposition in a community repository (e.g. GitHub). See the Nature Research guidelines for submitting code & software for further information.

Data

Policy information about availability of data

All manuscripts must include a data availability statement. This statement should provide the following information, where applicable:

- Accession codes, unique identifiers, or web links for publicly available datasets
- A list of figures that have associated raw data
- A description of any restrictions on data availability

The E. coli. transcriptional regulatory network data that support the findings of this study is available in the supplementary files of the following paper: X. Fang, A. Sastry, N. Mih, D. Kim, J. Tan, J. T. Yurkovich, C. J. Lloyd, Y. Gao, L. Yang, and B. O.Palsson, "Global transcriptional regulatory network for escherichia coli robustly connects gene ex-pression to transcription factor activities,"Proceedings of the National Academy of Sciences, vol. 114,no. 38, pp. 10286–10291, 2017. https://doi.org/10.1073/pnas.1702581114

Field-specific reporting

Please select the one below that is the best fit for your research. If you are not sure, read the appropriate sections before making your selection.

☒ Life sciences ☐ Behavioural & social sciences ☐ Ecological, evolutionary & environmental sciences

For a reference copy of the document with all sections, see nature.com/documents/nr-reporting-summary-flat.pdf

Life sciences study design

All studies must disclose on these points even when the disclosure is negative.

Sample size	Analysis based on simulation results have been done with sample size large enough to get a reasonably small standard error.
Data exclusions	No data exclusion has been done.
Replication	No new experimental findings has been reported in the manuscript. Reproducibility of simulation results is ensured through having multiple runs, clear representation of the methods used, and publicly shared codes.
Randomization	There was no grouping of data carried out. Randomization is not applicable to this study.
Blinding	Blinding is not relevant to this study, as the findings are based on a physical model described in the manuscript, and the simulations are analysis are based on this model.

Reporting for specific materials, systems and methods

We require information from authors about some types of materials, experimental systems and methods used in many studies. Here, indicate whether each material, system or method listed is relevant to your study. If you are not sure if a list item applies to your research, read the appropriate section before selecting a response.

Materials & experimental systems

n/a	Involved in the study	
☒	☐	Antibodies
☒	☐	Eukaryotic cell lines
☒	☐	Palaeontology and archaeology
☒	☐	Animals and other organisms
☒	☐	Human research participants
☒	☐	Clinical data
☒	☐	Dual use research of concern

Methods

n/a	Involved in the study	
☒	☐	ChIP-seq
☒	☐	Flow cytometry
☒	☐	MRI-based neuroimaging
